# Gut microbiota preserves bone mass through modulating the hyodeoxycholic acid–TGR5 axis: **Gut microbiota regulates bone mass**

**DOI:** 10.1080/19490976.2025.2593088

**Published:** 2025-12-02

**Authors:** Xuan-Qi Zheng, Jie Huang, Wan-Qiong Yuan, Tong Wu, Huan Wang, Hao Liu, Yun-Di Zhang, Jin-Wen He, Chen Huang, Chun-Li Song

**Affiliations:** a Department of Orthopaedics, Peking University Third Hospital, Beijing, China; b Center of Basic Medical Research, Institute of Medical Innovation and Research, Peking University Third Hospital, Beijing, China; c Beijing Key Laboratory of Spinal Disease Research, Beijing, China; d Engineering Research Center of Bone and Joint Precision Medicine, Beijing, China

**Keywords:** Osteoporosis, gut microbiota, bile acid, bone metabolism, TGR5

## Abstract

**Background:**

Osteoporosis is an age-related disease. The relationship between gut microbiota (GM) homeostasis and bone health is well established, but the mechanism of GM dysbiosis contributes to senile osteoporosis remains elusive. The objective of this study is to investigate the relationship between GM, bile acids (BAs) and their effects on bone mass.

**Results:**

16S rRNA sequencing and untargeted and targeted metabolomics revealed a reduction in microbial diversity, accompanied by the change of BA profile. In particular, the abundance of *Parabacteroides goldsteinii* and hyodeoxycholic acid (HDCA) in old mice were markedly decreased, compared with young mice. And there was a strong positive correlation between the abundance of *P. goldsteinii* and HDCA and bone mass. Further, our results demonstrated that old mice cohoused with young mice, with/without coprophagy prevention, were unable to alter the GM composition or reverse age-related bone loss. The transplantation of GM from young mice into old mice, rather than the transplantation of *P. goldsteinii* alone, reconstructed the GM of old mice and preserved bone mass by inhibiting bone resorption. Mechanistically, HDCA inhibits osteoclast maturation *in vitro* and exerts the bone protection effect *in vivo* through the activation of the G protein-coupled bile acid receptor (TGR5). HDCA treatment has been shown to result in the internalization of TGR5, thereby inhibiting the nuclear translocation of P65 *in vivo*. Knockout of TGR5 attenuated the effects of HDCA on bone microstructure, confirming these findings.

**Conclusions:**

This study identified the GM–HDCA–TGR5 axis is a key pathway that affects bone mass and targeted intervention of HDCA represents potential therapeutic option for osteoporosis.

## Graphical abstract

**Figure:**
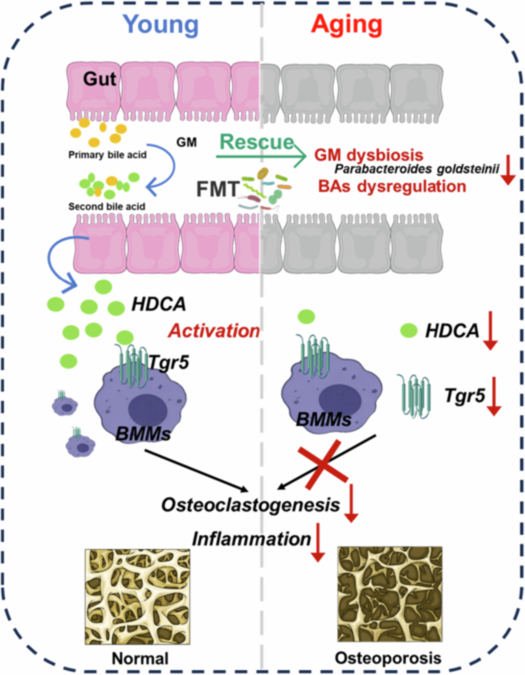

Schematic of hypotheses concerning the mechanisms underlying the role of the GM–bile acid–TGR5 axis in bone metabolism. GM and bile acid metabolism differed between young and old mice, *Parabacteroides goldsteinii* and HDCA may be the key factors. HDCA maintains bone mass by activating the bile acid receptor TGR5 of BMMs, and TGR5 deficiency accelerates bone loss.

## Introduction

Osteoporosis is a prevalent condition in the elderly population.^1^ Age is characterized by metabolic dysfunction,^2^ including intestinal microenvironment instability, bile acids (BAs) spectrum disorders, and systemic inflammation. The gut microbiota (GM) is involved in maintaining host metabolic homeostasis. The composition and abundance of the GM are influenced by a multitude of factors.^3^

The GM affects bone mass by influencing the host immune system, host metabolism, and metabolic products.^4^ The common metabolites of the GM include short-chain fatty acids, BAs, trimethylamine oxide, tryptophan, and other amino acids, which are closely related to bone metabolism.^3^ GM modulation is one of the potential ways to regulate bone metabolism. Among them, the cohousing model is a means of natural interventions that takes advantage of mammals' natural characteristics of coprophagy. Besides, fecal microbiota transplantation (FMT) is a means of artificial intervention in which a specific GM is transplanted into the recipient to reconstruct its gut microecology. It has been reported that cohousing with healthy mice prevents osteonecrosis of the femoral head in glucocorticoid-treated mice.^5^ Transplantation of GM from children prevents bone mass loss in mice with OVX-induced osteoporosis.^6^ It has been demonstrated that exercise has the capacity to reduce the elevated Firmicutes/Bacteroidetes ratio that has been observed in response to a high-fat diet, while concomitantly attenuating the process of bone loss.^7^ In addition, probiotics and prebiotics have been demonstrated to exert a bone-protective effect through the process of reshaping the gut microecology.^8^ However, more evidence is needed to elucidate the connections among aging, GM, and osteoporosis.

BAs are one of the important metabolites of the GM, and maintaining GM and BAs homeostasis is critical to host health. Recent studies have also linked bile acid metabolism to bone health. It was found that mice with postmenopausal osteoporosis^9^ or received a high-fat diet^10^ had abnormal BA metabolism accompanied by impaired bone metabolism. In addition, liver disease-induced osteoporosis and cholestatic osteoporosis are often associated with BA metabolism.^11-13^ In fact, BAs act as signaling molecules to activate the BA receptors FXR or TGR5 and subsequently affect host metabolism.^14^ We previously found that the GM was involved in estrogen deficiency-induced osteoporosis and is closely related to TGR5.^15^ However, the potential mechanisms of BA metabolism or BA receptor TGR5 in senile osteoporosis remain to be explored.

Here, we investigated the GM and metabolism difference between young and aged female mice. In order to further investigate the correlation between GM/BAs and senile osteoporosis, we focused on the changes of GM and BAs after cohousing and FMT treatment. We also identified HDCA–TGR5 axis as a key mechanism that regulates bone metabolism. Our current study provides the updated evidence that GM and BA homeostasis are potential targets for improving bone health in the elderly population.

## Method details

### Animals and experiments

Female C57BL/6J mice aged 2 months (young), 18 months (old), and 24 months (extremely old) were provided by the Laboratory Animal Center of Peking University (Beijing, China). TGR5-KO mice were provided by the Shanghai Southern Model Animal Center (Shanghai, China). The mice were kept in standard animal facilities in controlled temperatures (22 °C) and photoperiods (12 h of light and 12 h of darkness) and had free access to fresh water and food. The experiment was conducted under the Guidelines for Animal Experiments of Peking University Third Hospital, which was approved by the Institutional Animal Ethics Committee (M2021088).

**Figure 1.**
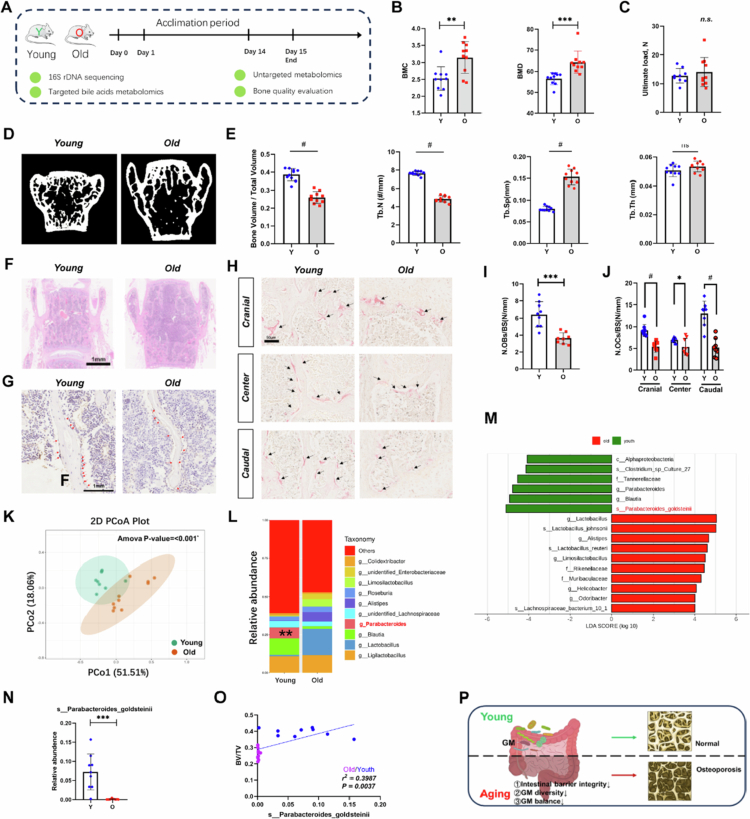
Gut microbial alterations in senile osteoporosis. A). Schematic diagram of 16S rRNA sequencing, untargeted metabolomics, targeted bile acid metabolomics and bone quality evaluation in 2-month-old C57BL/6J mice (*n* = 9) and 18-month-old C57BL/6J mice (*n* = 10). B). Quantitative analysis of the bone mineral content (BMC) and bone mineral density (BMD) of young and aged mice. C). Assessment of ultimate force in the vertebral body using compression testing. The error bars represent means ± SD. Values of *p* by two-tailed unpaired-samples *t*-test. D, E). Representative µCT images of the three-dimensional trabecular architecture (D) and quantitative analysis of changes in the trabecular microarchitecture (E) of the L5 vertebral body from young and old mice. F–J). Representative images of HE-stained (F) and OCN-stained sections (G) with quantification (I) of the number of osteoblasts (N. OBs) and TRAP-stained (H) sections with quantitation (J) of the number of osteoclasts (N. OCs) on trabecular bone surface (BS) in L5 vertebral body from young and aged mice. K). PLS–DA score plot of species abundance in samples from individuals with young mice (green points) and old mice (red points). Permutational multivariate analysis of variance with the Bray–Curtis distance metric was used to assess the significance of differences between the two groups (***p < 0.001). L). Composition of GM at the genus levels tested by 16S rRNA gene sequencing. As for genus, the proportion <1% occupancy is noted as the others. T-test analysis of differences in the relative abundance of species between groups based on OTU showed that *Parabacteroides* (the *p* value was determined by two-tailed Wilcoxon rank-sum test. The *q* value represents the FDR-adjusted *p* value; *q* < 0.01) was significantly different between the two groups. M). Taxa enriched in the young (green) and old (red) groups with LDA score ≥4 are indicated. N). Relative abundances of *P. goldsteinii* in young and old subjects. Statistical analysis was performed by two-tailed unpaired-samples *t*-test. *** *p* < 0.001. O). Pearson's correlation analyses were used to examine further relationships between the relative abundances of *P. goldsteinii* and BV/TV. P). Schematic diagram of the hypothesis that the GM affects bone mass through changes in GM and bacterial metabolites.

In experiment 1 (Figure 1A), we used ten 18-month-old C57BL/6 female mice as the old group and nine 2-month-old C57BL/6 female mice as the young group. At the end of the experiment, cecal contents were collected for untargeted metabolomics analysis, fresh feces were collected for 16S rRNA sequencing, blood samples were collected for targeted bile acid profile detection, and bone specimens were collected for bone mass assessment.

In experiment 2 (Figure 3A), nineteen 18-month-old female mice were randomly divided into two groups: the cohousing (Ch) group and the cohousing but coprophagy prevention group (Ch.CP group). Specifically, this consistent cohousing experiment was performed by consolidating old mice with young mice at a ratio of 1:5 in the same cage for 12 weeks, according to the reference with some modifications.^5^^,^^16^^,^^17^ The bottom of the cage of Ch.CP group was equipped with a bottom plate with round holes (with a 1 cm diameter) to separate the mouse feces and prevent the mice from coprophagy habits, while the Ch group was not given special treatment. After the intervention (cohousing treatment) was completed, the old mice (in Figure 3A, mice named Old^Ch^ corresponds to Ch group in Figure 3B; Old^Ch-CP^ group corresponds to Ch.CP group in Figure 3B) were tested.

**Figure 2.**
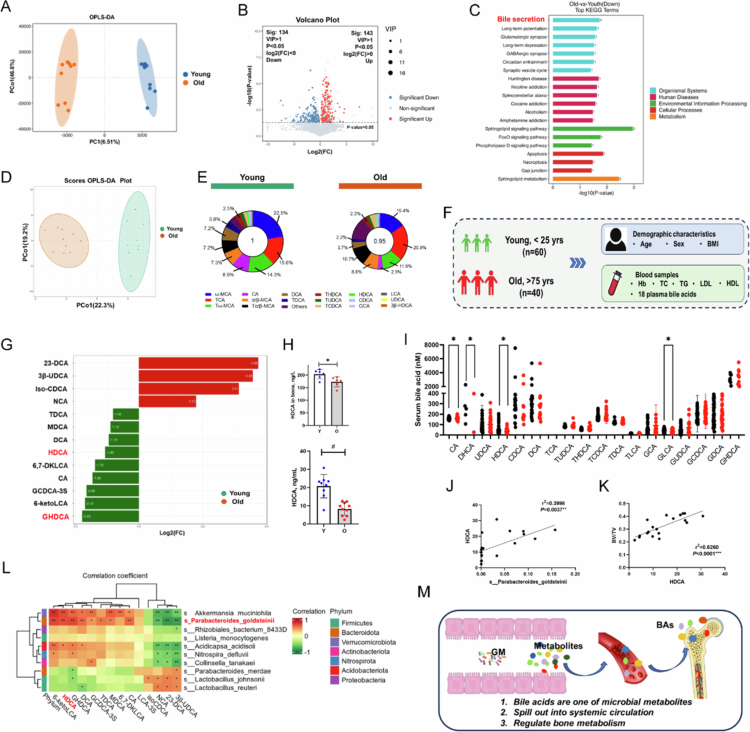
Metabolic profile of intestinal contents and blood alterations in senile osteoporosis. A). Untargeted metabolomics analysis was conducted for intestinal contents of young and old subjects. OPLS-DA score plot of bacterial metabolite abundance in samples from individuals with young mice (blue points) and old mice (orange points). B). Volcano plot showing differentially expressed metabolites between the young and old groups. C). Top 20 bar chart of KEGG pathway enrichment analysis between the young and old groups. D). Targeted bile acid metabolomics analysis was conducted for serum of young and old subjects. OPLS-DA score plot of bile acid abundance in samples from individuals with young mice (green points) and old mice (red points). E). The mean percentages of different bile acids in the respective groups are shown. The area of the whole ring represents the concentration of total bile acids. F). Schematic diagram of the cross-sectional study design. G). The bar chart shows the top 20 bile acids with different multiples. The horizontal coordinate is Log_2 FC of differential metabolite, and the vertical coordinate is the differential metabolite. Red represents upregulated differential metabolites, and green represents downregulated differential metabolites. H). ELISA analysis of HDCA in serum and bone. I). The levels of the serum bile acids of cholic acid (CA), dehydrocholic acid (DHCA), ursodeoxycholic acid (DHCA), ursodeoxycholic acid (HDCA), hyodeoxycholic acid (CDCA), deoxycholic acid (DCA), taurocholic acid (TCA), tauroursodeoxycholate (TUDCA), taurohyodeoxycholic acid (THDCA), taurochenodeoxycholic acid (TCDCA), taurodeoxycholic acid (TDCA), taurolithocholic acid (TLCA), glycocholic acid (GCA), glycolithocholic acid (GLCA), glycocholic acid (GUDCA), glycochenodeoxycholic acid (GCDCA), glycodeoxycholic acid (GDCA), and glycohyodeoxycholic acid (GHDCA) were detected in individuals. *p* values were determined by two-tailed Mann–Whitney *U*-test and the data are presented as medians with interquartile ranges. J). Pearson's correlation analyses were used to examine further relationships between the relative abundances of *P. goldsteinii* and HDCA. K). Pearson's correlation analyses were used to examine further relationships between the relative abundances of HDCA and BV/TV. L). Pearson's correlation hierarchical clustering heatmap of different microorganisms and different metabolites (*p < 0.05, **p < 0.01). M). Schematic diagram of the hypothesis that the change of GM causes the change of serum bile acid spectrum, which reaches the bone through the circulatory system and affects bone metabolism

In experiment 3 (Figure 4A), as for recipients, twenty 18-month-old mice were randomly assigned to 2 groups: the FMT group and the control group. Recipient mice should be single-housed to prevent cross-contamination and allow for individual fecal sampling. As for donors, 2-month-old female young C57BL/6J mice, feeding in the same nest (*n* = 20−30). Feeding was performed with standard feed without any special intervention. Fresh feces were collected at a fixed time (9 a.m.) on the day of FMT. One week before the experiment, all the mice were given a gavage antibiotic cocktail treatment (1  g/l ampicillin, 1 g/l metronidazole, 1 g/l neomycin sulfate, and 0.5 g/l vancomycin) once daily for 7 d to exhaust the host pre-existing GM. After microbiota deletion, the feces of all the mice were collected and used for metagenomics sequencing (named ABT group). Then ten mice were orally administered with 2 × 10^8^ CFUs of pooled GM of young mice (named FMT group), and another ten mice were given the same amount of PBS (named CON group). Intragastric administration was performed 3 times a week for 8 weeks. After continuous feeding for 4 weeks, samples were collected. All the mice were kept separately to rule out GM communication between individuals.

**Figure 3.**
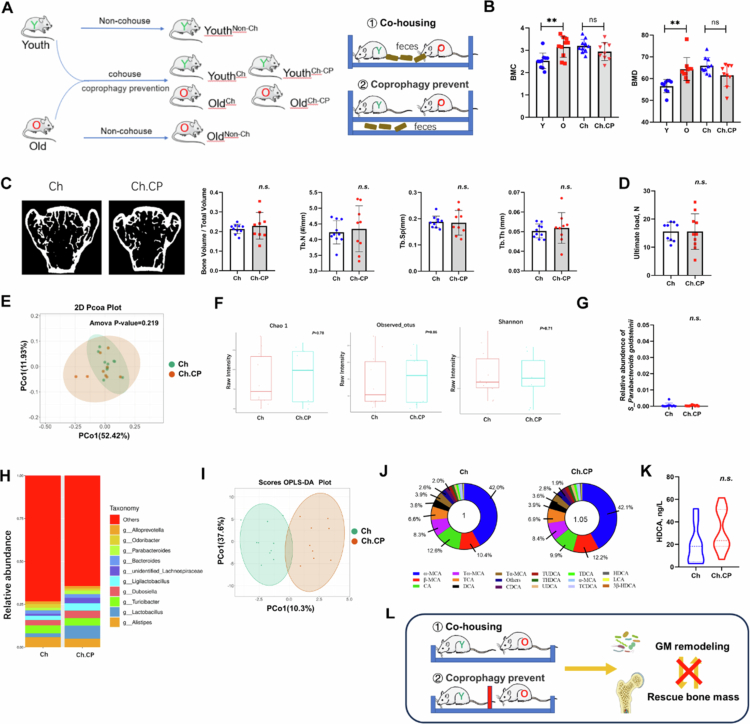
Co-housing or coprophagy prevention do not regulate bone metabolism by reshaping GM of old mice. A). Schematic diagram of the experimental design about cohousing model. Old mice cohoused with young mice were recognized as Ch group (*n* = 10); old mice cohoused with young mice but coprophagy prevention were recognized as Ch.CP group (*n* = 9). B). Quantitative analysis of BMC and BMD of young mice (without cohouse treatment), old mice (without cohouse treatment), Ch group and Ch.CP group. Error bars represent mean ± SD. Values of *p* by one-way ANOVA, followed by Tukey's post hoc test. C). Representative µCT images of the three-dimensional trabecular architecture and Quantitative analysis of changes in the trabecular microarchitecture of the L5 vertebral body from Ch group and Ch-CP group subjects. D). Assessment of ultimate force in vertebral body using compression testing. Error bars represent means ± SD. Values of *p* by two-tailed paired-samples *t*-test. E). OPLS-DA score plot of species abundance in samples from individuals with Ch group mice (green points) and Ch.CP group mice (red points). Permutational multivariate analysis of variance with the Bray–Curtis distance metric was used to assess the no significance of differences between the two groups (*p* = 0.219). F). Alpha diversity (Observed otus, Chao1 and Shannon index) of the Ch group and Ch.CP groups at the gene level. G). Relative abundances of *P. goldsteinii* in young and old subjects. Statistical analysis was performed by two-tailed unpaired-samples *t*-test (*p* > 0.05). H). Composition of GM at the genus levels tested by 16S rRNA gene sequencing. As for genus, the proportion < 1% occupancy is noted as others. The *p* value was determined by two-tailed Wilcoxon rank-sum test. The *q* value represents the FDR-adjusted *p* value; *q* > 0.05. I). Targeted bile acid metabolomics analysis conducted for serum of Ch group and Ch.CP group subjects. OPLS-DA score plot of bile acid abundance in samples from individuals with Ch group mice (green points) and Ch-CP group mice (red points). J). Mean percentages of different bile acids in the respective groups are shown. The area of the whole ring represents the concentration of the total bile acids. K). ELISA analysis of HDCA in serum of the Ch group and Ch.CP groups. L). Schematic diagram of the results shown that old mice cohoused with young mice with/without coprophagy prevention shown no significant in GM modulation and rescue bone loss.

In experiment 4 (Figure 6A), six 18-month-old mice were randomly assigned to 2 groups. The HDCA group mice were gavaged orally with 50 mg/kg/day of HDCA solution, while the control group mice were gavaged orally with the same amount of PBS. The BMD of the whole body was measured at 1 d (as the baseline data), 1 week and 4 weeks after treatment and then euthanized.

**Figure 4.**
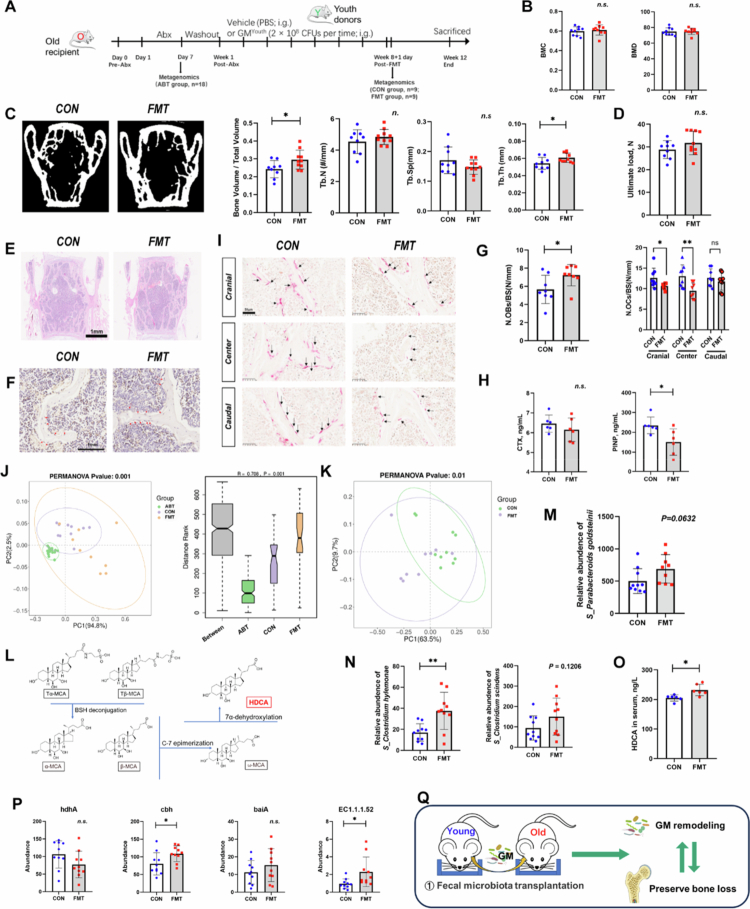
Structure of GM and the related metabolites as well as bone metabolism are changed in FMT mice. A). Timeline for the old mice received the treatment of FMT from young mice. After antibiotics treatment (Day 7), the feces of all the mice were collected and subjected to metagenomic sequencing, which was prescribed as the ABT group (*n* = 18). Then old mice with FMT treatment were recognized as FMT group (*n* = 9), and old mice without FMT treatment were recognized as control (CON group, *n* = 10). After treatment (week 8 + 1 d), the feces of the old mice in both the CON and FMT groups were collected and subjected to metagenomic sequencing. B). Quantitative analysis of BMC, BMD of CON group, and FMT group. C). Representative µCT images of the three-dimensional trabecular architecture and quantitative analysis of changes in the trabecular microarchitecture of the L5 vertebral body from CON group and FMT group subjects. D). Assessment of ultimate force in L5 vertebral body using compression testing. Error bars represent means ± SD. Values of *p* by two-tailed paired-samples *t*-test. E–I). Representative images of HE (E), OCN-stained sections (F) and TRAP-stained (I) sections with quantification (G) of the number of osteoblasts (N. OBs) and the number of osteoclasts (N. OCs) on trabecular bone surface (BS) in L5 vertebral body from CON group and FMT group subjects. H). ELISA analysis of the serum concentrations of bone turnover markers (PINP and CTX-I). J). Permutational multivariate analysis of variance with the Bray–Curtis distance metric was used to assess the significance of differences CON group mice (purple box), FMT group mice (yellow box) and ABT groups (green box) (*p* = 0.001). K). OPLS-DA score plot of species abundance in samples from individuals with CON group mice (green points) and FMT group mice (purple points). Permutational multivariate analysis of variance with the Bray–Curtis distance metric was used to assess the significance of differences between the two groups (*p* = 0.01). L). Synthetic pathways of HDCA in mice. In mice, α-MCA and β-MCA was deconjugated by BSH. ω-MCA was generated from α-MCA and β-MCA by C-7 epimerization, and HDCA was generated from ω-MCA by 7α-dehydroxylation. M). Relative abundances of *P. goldsteinii* in CON and FMT group subjects. Statistical analysis was performed by two-tailed paired-samples *t*-test (*p* = 0.0632). *N*). Relative abundances of the secondary BA-synthesizing bacteria *C. scindens* and *C. hylemonae*. O). ELISA analysis of HDCA in serum of the CON group and FMT groups. P). Boxplot of expression abundance of secondary bile acid production-related genes. Q). Schematic diagram of the results shown that old mice with FMT from young mice shown significant in GM modulation and rescue bone loss.

In experiment 5 (Figure 6H), six 2-month-old *Tgr5*^+/+^ mice and six 2-month-old *Tgr5*^−/^^−^ mice were all orally gavaged with 50 mg/kg/d of HDCA solution for 4 weeks and then euthanized.

Sequence files and metadata for all samples used in experiments 1, 2, and 3 have been deposited in Figshare (https://doi.org/10.6084/m9.figshare.28136381.v1).

### Specimen

After being anesthetized, the selected mice underwent abdominal massage, and then, the fresh feces were collected and stored in dry-ice storage equipment. After fresh feces were collected, the mice were euthanized, first, peripheral blood was collected, then the cadaver was dissected, and the cecal intestinal contents were collected in a sterile frozen storage tube. Finally, the spinal column, bilateral femur, and tibia were collected. The specimens were stored on dry ice or at −80 °C refrigerator.

## Microbiome analysis

### 16S rRNA sequencing and analysis

Fecal DNA was extracted using the Qiagen Kit. DNA quality was determined by agarose gel electrophoresis, and DNA quantity was determined by Nanodrop 2000 spectrophotometry. The V3-V4 region of the bacterial 16S rRNA gene was amplified by PCR using universal primers 343F and 798r. The purified amplicons were pooled in equal numbers for sequencing. The 16 s rRNA gene was sequenced by the Illumina MiSeq platform (MiSeq PE300, Illumina, San Diego, USA). Use UPARSE (version 7.1, http://drive5.com/uparse/) for high-quality sequence clustering to have 97% similarity OTU cutoff value, and use UCHIME to remove chimeras. Then, the OTU is divided into bounds, phyla, classes, orders, families, and genus levels, and finally the OTU table is created. The principal coordinates analysis (PCoA) based on weighted UniFrac was adopted to reflect the community similarity and overall differences of GM in each group, and the differences among the groups were analyzed by the Adonis test. 16S rRNA sequencing and analysis were supported by Metware Gene Technology Co., Ltd., Beijing, China.

### Metagenomic library preparation, sequencing, and analysis

Total DNA was extracted using the Qiagen Kit. The libraries were constructed using TruSeq Nano DNA LT Sample Preparation Kit (Illumina, San Diego, CA, USA). The libraries were conducted on an Illumina NovaSeq 6000 instrument, which yielded 150 base pair paired-end sequences. The raw sequences in the FastQ format were processed for quality trimming and filtering using Trimmomatic version 0.36. The cleaning processes were performed on the raw data. Assembly of the metagenome was carried out utilizing MEGAHIT version 1.1.2, following the acquisition of valid reads. The ORF prediction of the assembled scaffolds was conducted using Prodigal (v 2.6.3), and the resulting amino acid sequences were subsequently translated. Nonredundant gene sets were constructed for all the predicted genes using CDHIT (v 4.5.7). Sequence reads from each sample, free from contaminants, were matched to a unique gene collection (with 95% sequence similarity) utilizing the Bowtie2 (v 2.2.9) alignment tool, and the abundance of the gene in the corresponding sample was quantified. The metagenome sequencing and analysis were supported by Oebiothch Gene Technology Co., Ltd., Beijing, China.

## Metabolite measurement

### Untargeted metabolomics

A 60 mg sample was measured in a 1.5 mL centrifuge tube and triturated with a 5 mm tungsten carbide bead using a 60 Hz mill for 2 min. A solvent mixture consisting of methanol, acetonitrile, and water at a ratio of 2:2:1 (volume/volume/volume) was precooled to 1 mL and subjected to ultrasonication in an ice bath for 10 min. Following this, the solution was subsequently cooled to −40 °C for 12 h before being centrifuged at 4 °C at 12,000 rpm for 10 min to separate the supernatant.

Metabolomics were analyzed using UHPLC-Q-Exactive Plus MS. An ACQUITY UPLC HSS T3 column (2.1 × 100 mm), 1.8 μm column was used for separation. The flow rate was set at 0.35 mL/min, with the mobile phase comprising solvent A: 0.1% formic acid in water and solvent B: 100% acetonitrile. The gradient profile started at 5% solvent B for 2 min, linearly increased to 30% after 4 min, and to 100% after 14 min, where it was held for an additional 2 min before being ramped back down to 5% solvent B in 0.1 min, with a 1-min reequilibration phase. Mass spectrometry data were acquired in negative-ion and positive-ion modes of electrospray ionization, respectively. The instrument parameters were configured to cover a mass range of 70−1050 Da for MS acquisition. The mass resolution was set to 60,000 at m/z 200 for full MS scans and 15,000 for MS/MS scans at m/z 200.

### Targeted bile acids metabolomics

**Sample:** 50 µL of serum was taken, 10 μL of internal standard mixed working fluid with a concentration of 1  μg/mL and 200  μL of 20% methanol-acetonitrile were added. The samples were mixed at 2500 rpm for 10 min and then placed in a refrigerator at −20 °C for 10 min. The samples were centrifuged for 10 min at 12000 rpm at 4 °C. The supernatant was collected and concentrated in a concentrator. The concentrated solution was redissolved with 100 µL of 50% methanol-water and then transferred.

**LC/MS column:** An ACQUITY UPLC HSS T3 C18 (2.1 × 100 mm) 1.8 μm column was used for separation at a flow rate of 0.35  mL/min. The solvent system, A phase was ultrapure water (containing 0.01% acetic acid and 5 mmol/L ammonium acetate); B phase was acetonitrile (containing 0.01% acetic acid). The gradient starts at 5% B (0 min), increases to 95% B (10 min) and then returns to 5% B (12 min). The temperature was 40 °C, and the sample size was 3 μL.

**ESI-MS/MS:** Techniques such as electrospray ionization (ESI) in both positive and negative modes were employed to ionize bile acids for detection. The QTRAP®6500+ LC‒MS/MS system was used. The source temperature was set as 550 °C positive ion mode at an ion spray voltage of +5000 V, and negative ion mode at an ion spray voltage of −4500 V. The quantification of bile acids is achieved using multiple reaction monitoring (MRM).

### ELISA analysis of bile acid

The serum concentrations of hyodeoxycholic acid (HDCA, Cat. No. MM-928249O2), C-telopeptide of type Ⅰ collagen (CTX-I, Cat. No. MM-47165M2), procollagen type I N-Propeptide (PINP, Cat. No. MM-44814M2), interleukin-6 (IL-6, Cat. No. MM-0163M1), interleukin-1β (IL-1β, Cat. No. MM-0040M1) and tumor necrosis factor-α (TNF-α, Cat. No. MM-0132M1) were determined using commercially available immunoassay kits in accordance with the manufacturer's instructions (Meimian, Jiangsu, China).

## Bone quality evaluation

### DXA

After anesthetization, the mice were conducted BMD analysis with a small animal high-resolution collimator (UltraFocus DXA; Faxitron, Tucson, AZ, USA). At the same time, further automatic determination of fat content. Mice heads are excluded from the ROI. A technician with no knowledge of the experiment analyzed the DXA data.

### Micro-CT

Under the conditions of an effective pixel size of 8.82 μm, a current of 500 μA, a voltage of 80 keV and an exposure time of 1500 ms per 360 steps of rotation, the separated samples were scanned by the Siemens Inveon multimodal system. Fifty pieces were extended from the metaphysis to the distal end. Trabecular parameters, including the bone volume/tissue volume (BV/TV), trabecular thickness (Tb.Th), trabecular number (Tb.N), and trabecular spacing (Tb.Sp), were automatically determined from the region of interest (ROI).

### Biomechanics

Biomechanical tests were performed in a mechanical test system (MTS Landmark Systems, Eden Prairie, MN) with a load sensor of 100 N. The L4 vertebral body was isolated and used for compression tests to assess the strength of the vertebral body. The direction of the compression load test was cranial caudal direction of vertebral body. The compression loading speed was 1 mm/min until the vertebral body collapsed. The maximum force and stiffness are calculated according to the resulting force‒displacement curve.

The mechanical properties of the tibia and femur were evaluated by three-point bending mechanical tests. The tibia was placed laterally downward between the two supports, spaced 10 mm apart. The femur was also placed between the two scaffolds, and the distance between the scaffolds was adjusted to 8 mm. A 0.5 N preload was applied to the middle of the bone to hold it in place, and the central loading plate was driven at a speed of 1 mm/min until the bone broke. The maximum bending force was calculated according to the force‒displacement curve obtained.

### Histology

For histological evaluation, specimens were fixed in 4% paraformaldehyde for 24 h and decalcified in 0.5 ml of EDTA (pH = 7.4) at room temperature with continuous shaking for about 21 d. After dehydration, the specimens were embedded in paraffin. The numbers of corpora lutea and cystic follicles were counted. The results were confirmed by a pathologist. The sections were prepared, and the bone samples were longitudinally and serially sectioned into 5 μm sections and stained for hematoxylin and eosin (HE), OCN (Abcam, Cat. No. ab93876) and TRAP (Servicebio, Cat. G1050-50T) with a standard protocol as described previously. All of the sections were mounted onto a glass slide and observed by histomorphological examination under a light microscope (NIS-Elements 3.2, Nikon Eclipse 80i; Nikon). TRAP-positive cells were identified as osteoclasts. The OCN+ and TRAP+ areas on the trabecular bone surface were calculated in three sections per mouse using Image-Pro Plus version 6.0 (Media Cybernetics Inc., US).

### Constitutive internalization of TGR5

Immunofluorescence assay revealed the internalization of TGR5 in BMMs. Cell surface receptors of living BMMs were labeled for 30 min at 4 °C with an antibody directed against the TGR5. Subsequently, the cells were incubated for different time intervals at 37 °C in the presence or absence of 50 μm HDCA or 5 μm INT-777 (TGR5 agonist, as a positive control). Cell surface receptors were visualized by staining living cells with a secondary antibody coupled to a green fluorescent fluorophore, and internalized receptors were stained after fixation and permeabilization of the cells with secondary antibody carrying a red fluorescent fluorophore.

## RNA-Sequencing of HDCA-treated bone marrow-derived macrophages (BMMs)

### The extraction of primary BMMs

After being euthanized, 8-week-old C57BL/6 mice were sterilized with 75% ethanol and placed on a clean bench. The skin was cut, the femur was separated, and the muscle tissue was removed. Using scissors, the ends of the femur were cut, and then a 5 mL syringe was used to blow out the bone marrow from the femur using a cold medium. Then the bone marrow cells were filtered through a 70 μm cell filter to remove cell masses and bone fragments. The treated cell suspension was resuspended in α-MEM medium containing 10% FBS and 50 ng/mL M-CSF.

### RNA-Sequencing

RNA sequencing was used to analyze gene expression changes in BMMs treated with TBHP alone and TBHP combined with HDCA. RNA was extracted using Trizol reagent (Invitrogen). Transcriptome sequencing and analysis were conducted by Metware Gene Technology Co., Ltd., Beijing, China.

## Molecular docking

The optimized three-dimensional structures of the ligands (HDCA, compound CID: 5283820) were generated as SDF files from Pubchem (https://pubchem.ncbi.nlm.nih.gov/). The crystallographic data for the TGR5 (PDB ID: 7XTQ) protein structure were obtained from the RCSB Protein Data Bank (https://www.rcsb.org/) with a resolution of 3.20 Å. After the ligands were removed from PyMOL (v2.6.0, http://www.pymol.org), the downloaded 7XTQ.pdb proteins were used as input files for docking analysis. The ligand structures and protein molecules were imported into the Chai Discovery server (https://lab.chaidiscovery.com/), and the blind docking programs were performed as previously described.^18^

## Osteogenic differentiation and alizarin red staining

Human-derived bone marrow mesenchymal stem cells (HBMSCs, CP-H166, Pricella Biotechnology Co., Ltd., Wuhan, China) were plated in 24-well plates until 80% confluence. The BMSCs were then incubated with osteogenic differentiation medium and treated with HDCA at concentrations of 0, 5, 25, 50, 125, and 250 μM. The osteogenic differentiation medium was changed every 48 h.

After 14 d of differentiation culture, the cells were washed with ddH_2_0, fixed with 4% paraformaldehyde for 10 min, and then conducted alizarin red staining under instructions (Solarbio, Beijing, China). Then the stained cells were examined and the fractions of ARS-positive areas were calculated.

## Osteoclastogenic induction and TRAP staining

The BMMs were cultured in 48-well plates in α-MEM (HyClone, Logan, USA) supplemented with 50 ng/mL of receptor activator for nuclear factor κB ligand (RANKL; Novoprotein, Shanghai, China) and with HDCA concentrations of 0, 5, 25, 50, 125, and 250 μM. The Osteoclast differentiation medium was changed every 48 h.

After 7 d of differentiation culture, the cells were washed with ddH2O, fixed with 4% paraformaldehyde for 10 min, and then conducted TRAP staining, under instructions (Sigma‒Aldrich, USA). The areas of TRAP-positive osteoclasts were measured.

## Statistical analysis

All data were presented as mean ± SD. Under the assumptions of normality (Shapiro‒Wilk test, *p* > 0.05) and homogeneity of variances (Levene's test, *p* > 0.05), the Student's *t*-test was applied for two-group comparisons, and one-way ANOVA followed by Tukey's test was employed for multigroup comparisons. For all the experiments, *p* < 0.05 was considered to be significant and represented by *p < 0.05, **p < 0.01, ***p < 0.001, #*p* < 0.0001. In the context of omics data, the *p* values were adjusted to account for the error detection rate. This adjustment was facilitated by the Benjamini‒Hochberg false discovery rate program, which sets a threshold of *q* < 0.05 for determining statistical significance. GraphPad Prism software (version 6.01) was used for the above statistical analysis.

## Results

### Bone mass loss occurred due to aging

Whole-body BMC/BMD analysis revealed that the bone mass of young mice was significantly lower than that of old mice (Figure 1B). However, the micro-CT results revealed that the BV/TV and Tb.N of young mice were significantly greater than those of old mice, whereas the Tb.Sp of young mice was significantly lower than that of old mice in both the lumbar spine (Figure 1D,E) and the bilateral proximal tibia and bilateral distal femur (Figure S1D). The OCN staining and TRAP staining shown a decrease in the number of osteoblasts and osteoclasts (Figure 1F–J). In addition, there were significant differences in the ultimate force of the tibia and femur (Figure S1D) between the young group and the old group, but no differences in the vertebral body were observed (Figure 1C).

### The composition of the GM changed during aging

To confirm that mice also undergo changes in microbial community, 16S rRNA sequencing was conducted to characterize GM from both young and old mice. As shown, the old mouse group presented decreased α-diversity of the GM. The Chao 1 and observed Otu indices, which reflect the richness and diversity of the microbiota, were lower in the old group than in the young group, although the Shannon indices were not significantly different (Figure S1E). Principal coordinate analysis (PCoA) based on the weighted UniFrac distance index revealed significant differences in microbial composition between the two groups (multivariate analysis of variance: ANOSIM *p* < 0.001; *r*² = 0.802) (Figure 1K). According to the results of nonmetric multidimensional scaling, we also observed that β-diversity (Bray–Curtis dissimilarity) between samples increased in the old group, indicating that the microbiota structure in the old group was more heterogeneous than that in the young group (Figure S6A).

At the family level, the abundance of *Tannnerellaceae* was higher in the young group than the old group (Figure S6B). At the genus level, the old group was characterized by a decrease in secondary BA-producing genera, including *Blautia* and *Parabacteroides* (Figures 1L, S6B). At the species level, *P. goldsteinii* showed the most significant difference in abundance between the two groups, according to the results of the linear discriminant analysis effect size (LEfSe) method (Figure 1M), and the relative abundance of *P. goldsteinii* was also compared between the young and old groups (Figure 1N).

### Role of the GM in senile osteoporosis

The role of the GM in mice with senile osteoporosis was also explored. Notably, the relative abundance of *P. goldsteinii* was significantly positively correlated with the BV/TV (*p* = 0.0037, *r*² = 0.3987; Figure 1O). As the results, L5 BV/TV was positively correlated with changes in specific bacteria (at the phylum, class, order, and family levels), such as *Muribaculaceae* (family subgroup; *p* = 0.0476, *r*² = 0.2115), *Rikenellaceae* (family subgroup; *p* = 0.0175, *r*² = 0.2894), *Tannerellaceae* (family subgroup; *p* = 0.0037, *r*² = 0.3989), and *Parabacteroides* (genus subgroup; *p* = 0.0037, *r*² = 0.3989) (Figure S6C).

### Aberrant metabolic activities during aging

To further investigate the biological effects of the GM in the aging group, we performed an untargeted metabolomics analysis using cecal content samples. An orthogonal projection to latent structures (OPLS)-DA score plot of bile acid abundance shown that there was a significant different metabolic activities between the young and old groups (Figure 2A). The levels of 143 metabolites were significantly increased in the aging group compared with the young group, and the levels of 134 metabolites were significantly decreased (Figures 2B; S2 B,C).

**Figure 5.**
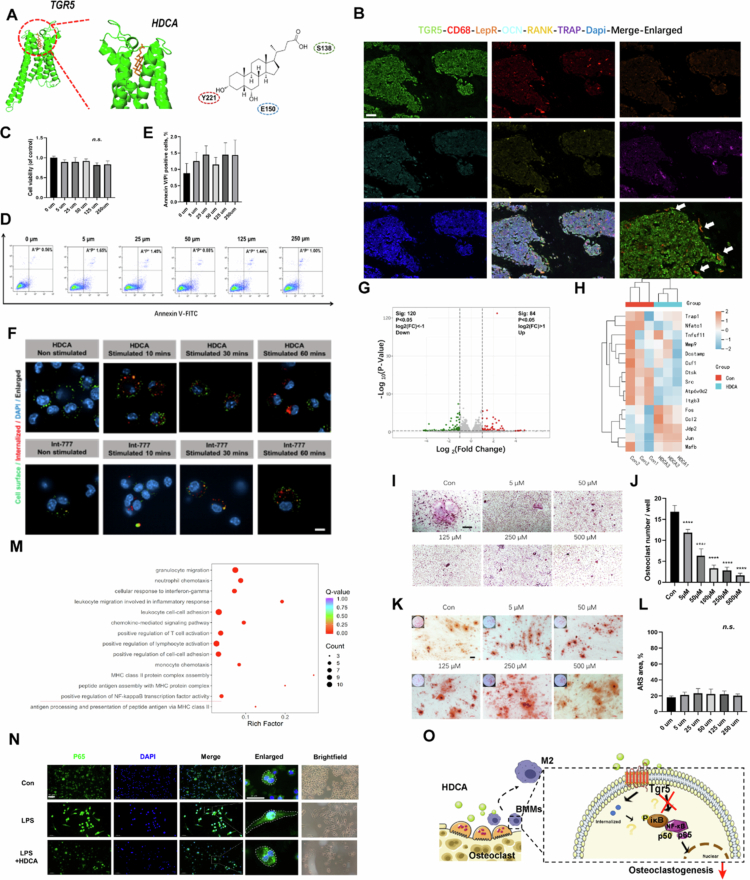
HDCA inhibits osteoclast maturation and macrophage antigen presentation via the TGR5 signaling pathway. A). Possible interactions between the HDCA (PubChem CID: 5283820) with GPBAR (PBD ID: 7xtq) were shown. B). Representative multicolor immunohistochemical staining for TGR5, CD68, LepR, OCN, RANK, and TRAP was performed at the distal metaphysis of the femur on aged mice. DAPI was used for nuclear counterstaining. Arrowheads indicate BMMs on the bone surface(scale bars = 50 µm). C). The cytotoxic effect of HDCA on BMMs was determined at various concentrations for 24 h using a CCK8 assay. D and E). Flow cytometry analysis and the quantification (E) of the proportion of Annexin V/PI positive macrophages under different treatments. *n* = 3 per group. F). Constitutive internalization of TGR5. Already after 10 min, small clusters of internalized receptors close to the cell membrane were observed. Activation of receptors with 100 μm HDCA or 5 μm INT-777 did not affect receptor internalization. G). Analysis of RNA-sequence of BMMs from different treatment groups after co-culture with TBHP for 2 h. Volcano plot (G) showing differentially expressed genes (DEGs) in the HDCA group compared to the control group. Genes with |log_2_FC| ≥ 1 & *p* < 0.05 are highlighted in green and red; colors denote downregulated and upregulated genes, respectively. H). Heatmap of genes related to osteoclastgenesis in the control and HDCA groups. I and J). Representative TRAP staining images of BMMs receiving different treatments (I) and quantification of osteoclast number per well in a 48-well plate (J). Scale bar: 50 µm. *n* = 3 per group. Data are presented as mean ± SD. K and L). Representative Alizarin Red S staining images of HBMSCs receiving different treatments (L) and quantification of osteoblast number per well in a 48-well plate (M). Scale bar: 100 µm. *n* = 3 per group. Data are presented as mean ± SD. M). GO database gene set enrichment analysis of regulatory gene pathways between HDCA group and control group. N). The p65 was detected by immunofluorescence combined with DAPI staining for nuclei. Scale bar: 50 μm. O). Schematic diagram of the hypothesis that the HDCA inhibits osteoclast maturation via activating the bile acid receptor TGR5.

Lolipopmap shows the 10 differential metabolites with the largest VIP value among the upregulated and downregulated differential metabolites respectively (Figure S2A). Further, according to the level-3 KEGG pathway enrichment analysis, there were 8 enriched metabolic pathways, which were related mainly to bile secretion, lipid metabolism, glucose metabolism and energy metabolism (Figure 2C). In addition, compared with the control group, gene set enrichment analysis (GSEA) showed that the “Bile secretion” were significantly inhibited in the old group. In contrast, “linoleic acid metabolism” was enhanced (Figure S2D).

### Serum BA spectrum changes as aging

In this study, 65 BAs were detected in the blood samples. The OPLS-DA score plot of bile acid abundance shown that there was a significant difference in the bile acid profile between the young and old groups (Figure 2D). The mean percentages of different bile acids in the respective groups are shown. The area of the whole ring represents the concentration of total bile acids (Figure 2E). Qualitative and quantitative analyses of the BA spectrum revealed that the serum levels of 23-DCA, 3β-UDCA, and isoCDCA were significantly increased in the aged group, whereas those of GHDCA and 6-ketoLCA were significantly decreased in this group (Figure S2E).

To investigate changes in the bile acid spectrum in aged individuals, 60 healthy control young donors and 40 aged persons were recruited. Body mass index, hemoglobin, and total cholesterol were not different between the two groups. But, old individuals had significantly higher triglyceride levels compared with healthy young controls (Table S1). Qualitative and quantitative analysis of bile acid species in serum indicated that HDCA were substantially reduced in the aged group compared with the young group (Figure 2F,I). The top 20 bile acids with different multiples were presented in the form of bar chart (Figure 2G). It is worth mentioning that hyocholic acid (HCA) species bile acids (including HDCA, and GHDCA) decreased in old mice. And GHDCA, a 6 glycine-conjugated bile acids, is one of the metabolites of HDCA.^19^ The HDCA level in bone was also tested by Elisa. The results shown that the HDCA level in aged mice was significantly lower than the young mice (Figure 2H).

### Correlation of GM, HDCA, and bone mess

Pearson correlation analysis of the GM and BA metabolism revealed a significant positive correlation between the relative abundance of *P. goldsteinii* and the serum HDCA level (*p* = 0.0037; *r*² = 0.3998) (Figure 2J,L). This finding is consistent with previous findings^20^ showing that *P. goldsteinii* is associated with BA metabolism. Furthermore, the HDCA level in mouse blood was related to the BV/TV of the lumbar vertebra (*p* < 0.0001; *r*² = 0.6260) (Figure 2K).

### Cohousing did not exert effect on bone

To examine the effects of cohousing and coprophagy behavior on GM and bone mass, the old mice were cohoused with young mice with or without coprophagy prevention (Figure 3A). The total BMD was lower in the Ch.CP group than in the Ch group, but the difference between the two groups was not statistically significant (Figure 3B), and there was no difference in the lumbar bone microstructure between the two groups (Figure 3C). Similar to the results for the lumbar vertebrae, the BV/TV, Tb.Th, Tb.N, and Tb.Sp of both the bilateral distal femurs and the proximal tibia did not show similar changes in bone microstructure (Figure S3A). In addition, there were no significant differences in the ultimate force of the L5 vertebral body (Figure 3D) or of the tibia and femur (Figure S3B) between the Ch group and the Ch.CP group.

### Cohousing did not reshape GM composition

The GM and BA profiles were determined for the Ch group and Ch.CP group. PCoA based on the weighted UniFrac distance index revealed no significant differences in microbial community structure between the two groups (Figure 3E; ANOSIM, *p* = 0.219). The Chao 1 and Shannon indices were not significantly different between the Ch group and the Ch.CP group (Figure 3F). The relative abundances of microbes at different classification levels are shown in Figure 3H and Figure S3C. There were also no significant differences in the relative abundance of *P. goldsteinii* between the young and old groups (Figure 3G).

### Cohousing did not change the BA metabolism profile

The OPLS-DA score plot of bile acid abundance shown that there was no significant difference between the two groups (Figure 3I). The mean percentages of different bile acids in the respective groups are shown. The area of the whole ring represents the concentration of total bile acids (Figure 3J). Qualitative and quantitative analyses of the BA spectrum revealed that the serum levels of 3β-CA (*p* = 0.110), β-GCA (*p* = 0.069), and 7,12-DKLCA (*p* = 0.106) were increased in the Ch.CP group, while the expression of GDCA was decreased in the Ch.CP group (Figure S3D). There were also no significant difference in the serum HDCA level between the two groups (Figure 3K).

### GM from young mice prevents bone loss and alter bone metabolism in aged mice

To explore whether the GM can prevent bone loss of osteoporotic recipients, we established an animal model of senile osteoporosis in conventionally raised 18-month-old C57BL/6 mice. We harvested GM samples from the same litter of 2-month-old young mice. We mixed the different donor-derived GM equally and investigated the effects of the GM mixture on bone metabolism (Figure 4A).

After 8 weeks, three times a week of oral administration of the pooled GM, there was no difference in whole-body BMD between the FMT recipient group and the control group (Figure 4B). Micro-CT analysis of lumber revealed that the BV/TV and Tb.Th of the FMT recipient group were significantly greater than those of the control group (Figure 4C).

Consistent with the bone microstructures, lumber compression testing indicated that the FMT mice had higher average values of ultimate force in L5 vertebral body compared with the control group, but only by trend (Figure 4D). These findings suggest that colonization with the pooled GM may block the reduction of maximum compressed load of the lumber in senile mice. We then tested the impacts of FMT on osteogenesis and osteoclastogenesis in aged mice. The microstructure analysis and the ultimate force of the tibia and femur showed no significant differences between the young group and the old group (Figure S4A–E)

OCN immunohistochemical staining showed a greater number of osteoblasts on the trabecular bone surface in the FMT mice compared with control aged mice (Figure 4F,G). Tartrate resistant acid phosphatase (TRAP) staining indicated that aging induced marked increases in the number and size of osteoclasts, but the effects were entirely reversed by colonization with GM from young mice. Consistently, enzyme-linked immunosorbent assay (ELISA) revealed that colonization with GM from young mice further augmented the serum level of C-terminal cross-linked peptide (CTX-I) between the CON and FMT groups (Figure 4H–J).

### GM changed in heterochronic FMT mice

To study the impact of antibiotic treatment/FMT on GM reconstruction, we collected stool specimens from mice in ABT group, CON group, and FMT group (Methods, experiment 4) and evaluated the microbial composition and function in these specimens by high-throughput sequencing of Metagenomics. The composition of the identified microbiota was profiled at the levels of phylum, class, order, family, genus, and species.

PCoA, ANOSIM and alpha diversity analyses showed significant differences among ABT, CON, and FMT groups. These findings indicate that after antibiotic cocktail treatment for 7 d, the original GM in aged mice were deleted. In addition, the degree of recovery of the gut microecology was also different in the old mice that were given PBS (CON group) or GM from young mice (FMT group) (Figures 4J, S4G).

To further find out the beneficial microbe that mediates the bone beneficial effects of FMT, CON group, and FMT group were compared. PCoA analysis showed significant differences between the two groups at the species level (Figure 4K, *p* = 0.01), and alpha diversity analysis showed that the *observed otus* index were different between the two groups (*p* = 0.04) (Figure S4H). It is suggested that FMT significantly changes in the GM of the recipients, and the number of species observed in the FMT group was significantly greater than that CON group, implying that the GM from young mouse donors successfully colonized in the GM of aged recipients and enriched the characteristic species GM of aged mice. At the phylum level, the abundances of *Bacillota*, *Thermodesulfobacteriota* and *Campylobacterota* in the FMT group were significantly greater than those in the ABT group. The abundance of *Uroviricota* decreased significantly. At the genus level, the abundances of *Oscillibacter* and *Eubacterium* in the FMT group were significantly greater than those in the CON group. The abundances of *Duncaniella*, *Paramuribaculum* and *Barnesiella* decreased significantly (data not shown). The heatmap shows the top 50 genus abundance in the samples from individuals with CON group and FMT group mice (Figure S4I). In addition, we found that the abundance of *P. goldsteinii* was higher in the FMT recipient group, implying that *P. goldsteinii* in aged mice was recovered (*p* = 0.0632, Figure 4M).

Overall, our results show that heterochronous FMT leads to significant alterations in the recipient microbiome structure, with an enrichment of bacterial species specific to young donors.

### The function of the GM changed in heterochronic FMT mice

Furthermore, we analyzed the abundance of microbial metabolic pathways and the changes in the potential microbial functional spectrum of receptors to clarify the effects of FMT on GM function. Alterations in microbiome-related metabolites is one of the ways through which the microbiome influences host function, including potential physiological and metabolic effects on bone mass. We found that lipid metabolism was downregulated among the enriched KEGG pathways (Figure S6E), which means that the abnormal lipid metabolism of the elderly mice (Figures 2A–C & S2A–C) was reversed by FMT.

To assess the changes in bile acid metabolism after FMT, we examined the abundances of the secondary BA-synthesizing bacteria *Clostridium scindens* and *Clostridium hylemonae* (Figure 4N). According to the synthetic pathways of HDCA in mice, HDCA was generated from ω-MCA by 7α-dehydroxylation (Figure 4L). The expression abundance of secondary bile acid production-related genes were also increased after FMT (Figure 4P). Besides, the level of HDCA was higher in the FMT recipient group (Figure 4O), the recovery of secondary BAs indicated the recovery of BA metabolism in the body.

### Detecting cavities and molecular docking

Although HDCA has a potential bone-protective effect, there remains a significant gap in understanding. We intended to reveal its interaction mechanism with the bile acid receptor TGR5 through molecular docking. The structure of TGR5 (also known as Gpbar-1) is the basis for the recognition and binding of bile acids.^21^ To further verify the hypothesis of the effect of HDCA on bone mass, we first used Chai-1 software to predict the unique structural features of TGR5 involved in bile acid recognition and allosteric effects. The Chai-1, a multimodal foundation model for molecular structure prediction produced by Chai Discovery, was used to search for cavities. Below are the crystal structure results of GPBAR, whose cavities and the possible interactions between HDCA and TGR5 are highlighted in Figure 5A.

**Figure 6.**
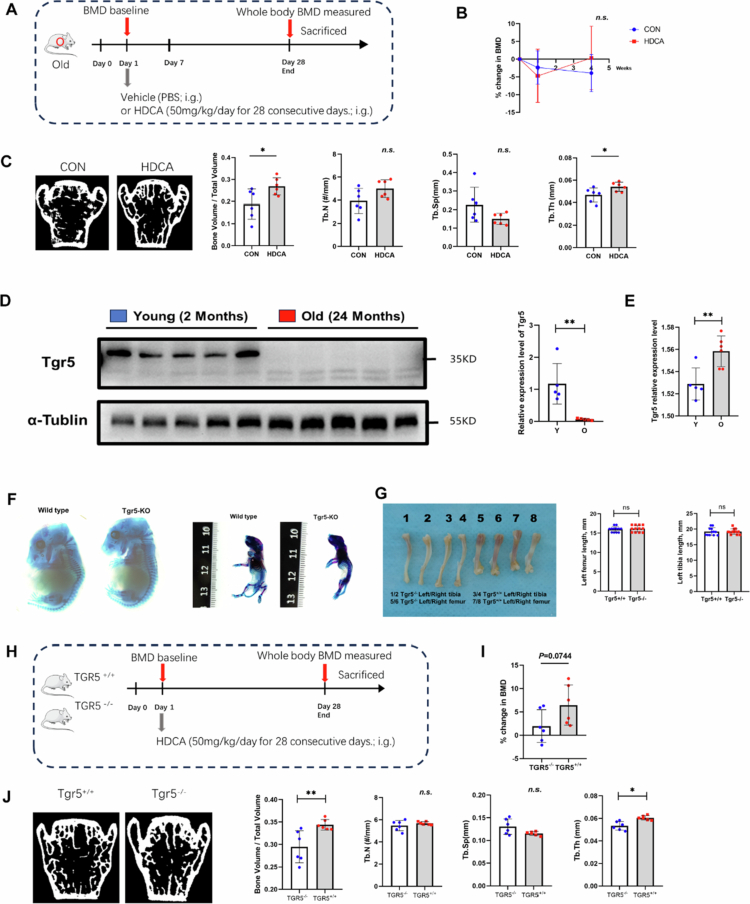
The bone protective effect of HDCA depends on the expression of TGR5. A). Timeline for the old mice received the treatment of HDCA. Old mice with HDCA treatment were recognized as HDCA group, and old mice without HDCA treatment were recognized as control (CON group). At the 1 d, 7 d, and 28 d after treatment, the whole-body BMD was detected. *N* = 3. B). Dynamic changes in whole-body BMD relative to the values at 1 d following HDCA treatment. Values of *p* were shown and compared between HDCA group at 4 weeks. Error bars represent mean ± SD, followed by two-tailed unpaired-samples *t*-test. C). Representative µCT images of the three-dimensional trabecular architecture and quantitative analysis of changes in the trabecular microarchitecture of the L5 vertebral body from CON group and HDCA group subjects. D). Protein expression and relative quantitation of TGR5 analyzed by western blot in the femur bone tissues of 24-months extreme old female wildtype mice and 2-months old female wildtype mice as control (*n* = 5). E). Quantitative real-time PCR analyses to determine the mRNA expression levels of TGR*5* in the femur bone tissues of 24-months old female wildtype mice and 2-months old female wildtype mice as control. F) Representative images of Alcian Red and Alcian Blue double stain of 18 dpc and P7 skeletons. Scale bar: 50 µm. G). Representative images of femur and tibia specimen obtained from 2-months old wildtype or TGR5 knockout mice and quantitative analyses showing the femur and tibia specimen length in mice. H). Timeline for the old mice received the treatment of HDCA. Wildtype 2-months old mice with HDCA treatment were recognized as *Tgr5*^+/+^ group, and *Tgr5*^−/−^ 2-months old mice with HDCA treatment were recognized as *Tgr5*^−/−^ group. At the 1 d, 28 d after treatment, the whole-body BMD was detected. *N* = 6 mice per group. I). Dynamic changes in whole-body BMD relative to the values at 1 d following HDCA treatment. Values of *p* were shown and compared between HDCA group at 4 weeks (*n* = 6 mice per group). Error bars represent mean ± SD, followed by two-tailed unpaired-samples *t*-test. J). Representative µCT images of the three-dimensional trabecular architecture and quantitative analysis of changes in the trabecular microarchitecture of the L5 vertebral body from *Tgr5*^+/+^ group and *Tgr5*^−/−^ group subjects.

### HDCA reduces the inflammation and MHC-related gene expression

To identify the target cells that the HDCA–TGR5 axis might act on, we performed multicolor immunofluorescence staining on bone tissue. The results showed that TGR5 was widely expressed in bone tissue, among which CD68 and TGR5 had obvious co-localization performance. This suggests that bone marrow macrophages may be potential target cells (Figure 5B). The BMMs were extracted to examine the effect of HDCA on inflammation by RNA-sequence. First, we examined the potential cytotoxicity of HDCA on BMMs by increasing the concentration of HDCA (0, 5, 25, 50, 125, and 250 μM) for 24 h, followed by CCK-8 analysis. HDCA shown no meaningfully reduced the cell viability at a concentration of 250 μM after 24 h of treatment. These findings suggest that HDCA is not cytotoxic to BMMs at concentrations below 250  μM before 24 h (Figure 5C). Flow cytometry analysis also shown no obvious improvement of the proportion of Annexin V/PI-positive macrophages under different treatments (Figure 5D). The analysis of dual staining with fluorescent Annexin V and PI shown that the proportion of early apoptotic cells (Annexin V-positive/PI-negative), late apoptotic cells (Annexin V-negative/PI positive) and apoptotic cells (Annexin V positive/PI positive) increased in a concentration-dependent manner (following a 24-h treatment period) (Figure S5A,C). However, when the cells were treated with 50 µm HDCA for 6 h, 12 h, and 24 h, no statistical difference was observed (Figure S5B,D).

### HDCA prevent osteoclastgenesis rather than osteogenic effect

BMMs treated with complete medium with increasing concentrations of HDCA (0, 5, 25, 50, 125, and 250  μM) formed less TRAP + osteoclasts than those treated with complete medium without HDCA, and these effects were further augmented with the increasing concentrations (Figure 5I,J). According to the results of TRAP staining, the addition of HDCA could inhibit the maturation of osteoclasts. The osteogenesis-related genes were also analyzed. HDCA significantly reduced the gene expression of Trap1, Nfatc1, MMP9, and Ctsk compared with the control group (Figure 5H).

HBMSC were treated with complete medium with increasing concentrations of HDCA (0, 5, 25, 50, 125, and 250  μM). Alizarin red staining was used to evaluate the osteogenic effect of HDCA, and no statistical difference was found among the groups (Figure 5K,L).

### HDCA activates TGR5 and promotes the internalization of TGR5 and inhibits the nuclear translocation of P65

To understand the potential mechanisms by which HDCA affects bone metabolism. The BMMs under TBHP-stimulated with HDCA treatment group were the HDCA group, and the BMMs without HDCA treatment were the con group. We used RNA-seq to investigate differentially expressed genes (DEGs, |log_2_FC| ≥ 1 & *p* < 0.05) between BMMs in different treatment groups (Figure S5E). As shown in the volcano plot (Figure 5G), 84 genes were upregulated and 120 genes were downregulated in expression of HDCA group compared to the con group.

We analyzed the above two differentially expressed genomes based on GO pathway enrichment and KEGG pathway enrichment. GO pathway analysis results shown that “positive regulation of NF-κB transcription factor activity pathway” was downgraded (Figure 5M). Then the P65 was detected by immunofluorescence combined with DAPI staining for nuclei. As shown in Figure 5N, under stimulation of LPS (simulating systemic inflammation of aging), the shape of BMMs changed from round or oval with short protrusions (M0) to dispersed cells with more pseudopods (classically activated M1); at the same time, the nuclear translocation of P65 increased. However, HDCA intervention transformed BMMs into a fried egg form (alternatively activated M2) and reversed the nuclear translocation of P65. Besides, the apoptosis- and inflammation-related genes were also analyzed (Figure S5F,G).

### Loss of TGR5 eliminates the bone-protective effect of HDCA in aged mice

The dynamic whole-body BMD results revealed that the mice in the HDCA group exhibited no significant difference of bone loss than those in the control group (Figure 6B). In contrast, the micro-CT results suggested that HDCA improved the bone microstructure of aged mice (Figure 6C).

We then compared the expression of TGR5 in the bone of young (2-month-old) mice and extremely aging (24 month-old) mice. The western blot results shown a significant decrease in TGR5 in extremely aging mice (Figure 6D). But, the gene expression of TGR5 shown an opposite trend (Figure 6E), that suggested posttranscriptional modifications may occur with the aging process.

Besides, the bone development between *Tgr5*^*+/+*^ mice and *Tgr5*^*−/−*^ mice during the fetal or early childhood period was compared, but there were no significant differences in the development of the limb skeleton (Figure 6F,G). To evaluate the effect of TGR5 in the bone-protective effect of HDCA (Figure 6H), we compared the effects of HDCA on *Tgr5*^*+/+*^ mice and *Tgr5*^*−/−*^ mice for 4 weeks and found that the increase in BMD in wild-type mice was greater than that in TGR5 knockout mice (*p* = 0.0744; Figure 5I). The micro-CT results revealed that the BV/TV and Tb.Th of the *Tgr5*^*+/+*^ mouse group were greater than those of the *Tgr5*^*−/−*^ mouse group, but there was no significant difference in the Tb.N and Tb.SP values between the two groups (Figure 5J). These findings suggest that the secondary bile acid HDCA may play a bone-protective role through the BA receptor TGR5.

## Discussion

GM metabolism provided a new way to explain the pathogenesis of osteoporosis. Here, we found that the GM and bile acid metabolism differed between young and old mice, *P. goldsteinii* and HDCA may be the key factors. Second, we confirmed the beneficial role of FMT, rather than cohousing, in rescue senile osteoporosis. Finally, we found that HDCA maintains bone mass by activating the BA receptor TGR5, whereas TGR5 deficiency accelerates bone loss. Our study suggests a new model of GM influence on bone metabolism through the BA–TGR5 axis.

BA metabolism has also been linked to aging. Certain BAs, such as isoallolithocholic acid (isoallo-LCA), which kills the harmful gut bacterium *Clostridium difficile*, are common in people over the age of 100 y,^22^ antimicrobial effects are one way in which these BAs may help with the immune response and promote health. The nontargeted metabolomics analysis of this study revealed significant lipid metabolism disorders in elderly individuals. Bile acids participate in the emulsification, digestion, and absorption of dietary lipids. Consequently, this finding suggests a potential link between osteoporosis and lipid metabolism disorders during the aging process, with the metabolism of bile acids potentially serving as a crucial connection point.

Bile acids, as vital signaling molecules, play a pivotal role in regulating metabolism. Different BAs have different effects. Deoxycholic acid (DCA) and cholic acid (CA) are known to cause DNA damage^23^ and thus affect mitochondrial function.^24^ Changes in the BA spectrum are also associated with the onset of Alzheimer's disease^25^ and Parkinson's disease.^26^ However, the administration of TUDCA improves age-related metabolic abnormalities and Aβ accumulation.^27^ TUDCA has also been shown to be neuroprotective in chronic mouse models of Parkinson's disease.^28^

The GM is transmitted between generations through a variety of vertical and/or horizontal patterns.^29^ Many small mammals have developed coprophagic habits or fecal ingestion behaviors to meet nutritional requirements and maintain stable diversity of the GM.^30^ Therefore, coprophagy is one of the bases for the development of cohousing animal models. Preventing coprophagy blocks the communication and transfer of the GM between individuals.^17^

It has been demonstrated that the cohabitation of young mice with aged mice has the effect of reversing the aging-related inflammation and rescuing the systemic BA homeostasis disorders observed in aged mice.^16^ However, in this study, although the bone mass of old mice cohousing with young mice was greater than that of old mice that were not cohoused (Ch group vs. old group), the difference was not statistically significant. Besides, the bone microstructure of the lumbar vertebrae and limbs shown no significant difference with or without coprophagy prevention (Ch vs. Ch.CP group). Furthermore, it has been demonstrated that the cohabitation of normal mice can mitigate femoral head necrosis in glucocorticoid-induced osteoporosis.^5^ This phenomenon is attributed to the alteration of bone metabolism by the GM, which is mediated by exosomes.

The transplantation of the GM from young rats has been demonstrated to alleviate senile osteoporosis. This is achieved by restoring the GM composition and gut barrier function.^31^ Furthermore, FMT from healthy mice has been demonstrated to prevent OVX-induced bone loss by modulating the GM and metabolic function.^32^ Nevertheless, the consequences of transferring the GM from aged mice to younger mice remain uncertain. It has been reported that the transplantation of the GM from old mice to young mice does not result in a reduction in bone mass or bone strength.^31^ However, an alternative study reached the opposite conclusion, namely, that the GM from aged mice induces osteoporosis in young recipient mice.^33^ The discrepancy between the two experiments was the intervention used for the control group (GM from young donor mice^31^ vs*.* saline.^33^

FMT has been demonstrated to extend the healthy lifespan in a pregeroid mouse model, the recovery of secondary BA levels represents a potential mechanism underlying the beneficial effects of restoration of healthy gut microecology.^34^ Melatonin has been demonstrated to reduce *Campylobacter jejuni*-induced deconjugation of TUDCA and GUDCA, thereby suppressing age-related high levels of FXR expression in the liver. This, in turn, has been shown to result in a further reduction in liver trimethylamine-*N*-oxide production, which in turn alleviates hepatic lipid metabolism abnormalities.^35^ This finding indicates that, in addition to monitoring the impact of FMT, it is essential to consider the influence of the GM on downstream metabolites.

Unexpectedly, the GM of aged mice that received FMT did not mirror that of the young mouse donors. The results of another study corroborate these findings^36^; the study demonstrated that although there was a notable compositional alteration in the microbiome of older mice following the transplantation of a younger donor microbiome, the characteristics of the older mice after this change were distinct from those of the younger mice. One potential explanation for this is that the transplanted microbiota from a younger donor is more susceptible to influence by the recipient's internal environment. Additionally, prolonged intensive feeding (such as oral gavage) may also serve as a significant stressor for aged mice.

Furthermore, it should be noted that this study did not involve the transplantation of individual strains, despite the suggestion that *P. goldsteinii* may represent a promising bone-conscious strain. It may be advantageous to restore the internal microecology as a whole rather than applying specific microbes in isolation. The supplementation of a single species is not an optimal approach for the restoration of GM diversity and the maintenance of intestinal microecological balance.^37^ In contrast to the transplantation of individual microbes, the holistic transfer of the GM from a healthy donor has the potential to restore and stabilize the recipient's intestinal microecological balance, thereby exerting a beneficial influence on the treatment of intestinal and external diseases, including cancer.^37^

In other words, the observed diversity, including contrasting results, following GM transplantation may be attributed to differences among flora donors, the specific characteristics of the aged hosts, or variations in the stability of bacterial strain colonization following allogeneic GM transplantation.^38-40^

It is also conceivable that the reduction of aging-induced chronic inflammation may represent another mechanism for GM. For example, FMT has been demonstrated to reduce inflammatory signaling and restore the viability of aging hematopoietic stem cells.^41^ Furthermore, FMT has been demonstrated to reduce stress, age-associated central nervous system inflammation and retinal inflammation.^36^ In addition, alterations in dietary habits,^42^ the utilization of pharmacological agents^43^ and caloric restrictions^44^ can alter the structure of the GM while reducing the level of body inflammation, thus enhancing the health of elderly individuals. Our previous research demonstrated that estrogen deficiency results in bone loss via the GM, with TGR5 and inflammatory factors identified as pivotal regulators of bone metabolism.^15^ This study further revealed that HDCA induces M2-type BMMs by activating TGR5 of BMMs, inhibiting the nuclear translocation of NF-κB, reducing MHC expression, and promoting CD163 expression. Furthermore, the results obtained from TGR5-knockout mice demonstrated that HDCA is dependent on TGR5 for its bone protective effects.

## Shortcomings

The present study focused exclusively on aged female mice. However, it should be noted that the effects of aging and the gut flora on individuals are sex specific. Consequently, osteoporosis in aged men should also be considered. Furthermore, although our previous research examined the impact of estrogen decline (via ovariectomy) on the GM and bone mass separately, this study employed aged female mice, characterized by both estrogen decline and aging. Consequently, the independent or additive effects of estrogen and aging warrant further investigation. The prevention of osteoporosis necessitates the implementation of early intervention strategies. Consequently, the exploration of preventive measures for middle-aged mice is a crucial avenue for further investigation.

## Conclusion

The aging process is associated with alterations in the composition of the GM, which in turn has been linked to reduced bone mass. This observation has led to the suggestion that the "gut‒bone axis" may represent a possible novel factor influencing bone metabolism. Further study yielded additional evidence that alterations in the GM reduce bone loss and may play a role in regulating bone metabolism through the BAs-TGR5 pathway. In conclusion, our findings contribute to the understanding of how to enhance gut microecology and promote bone health.

## Supplementary Material

Supplementary material Supplementary material.

## Data Availability

Sequence files and metadata for all samples used in experiment 1, 2, and 3 have been deposited in Figshare (https://doi.org/10.6084/m9.figshare.28136381.v1).
