# Supplementary material for: Gut microbiota preserves bone mass through modulating the hyodeoxycholic acid–TGR5 axis: Gut microbiota regulates bone mass
Source: Gut Microbes. 2025 Dec 2;17(1):2593088. doi: 10.1080/19490976.2025.2593088 (PMC12688228; doi:10.1080/19490976.2025.2593088)
Supplement: Supplementary material [file KGMI_A_2593088_SM4167.docx]

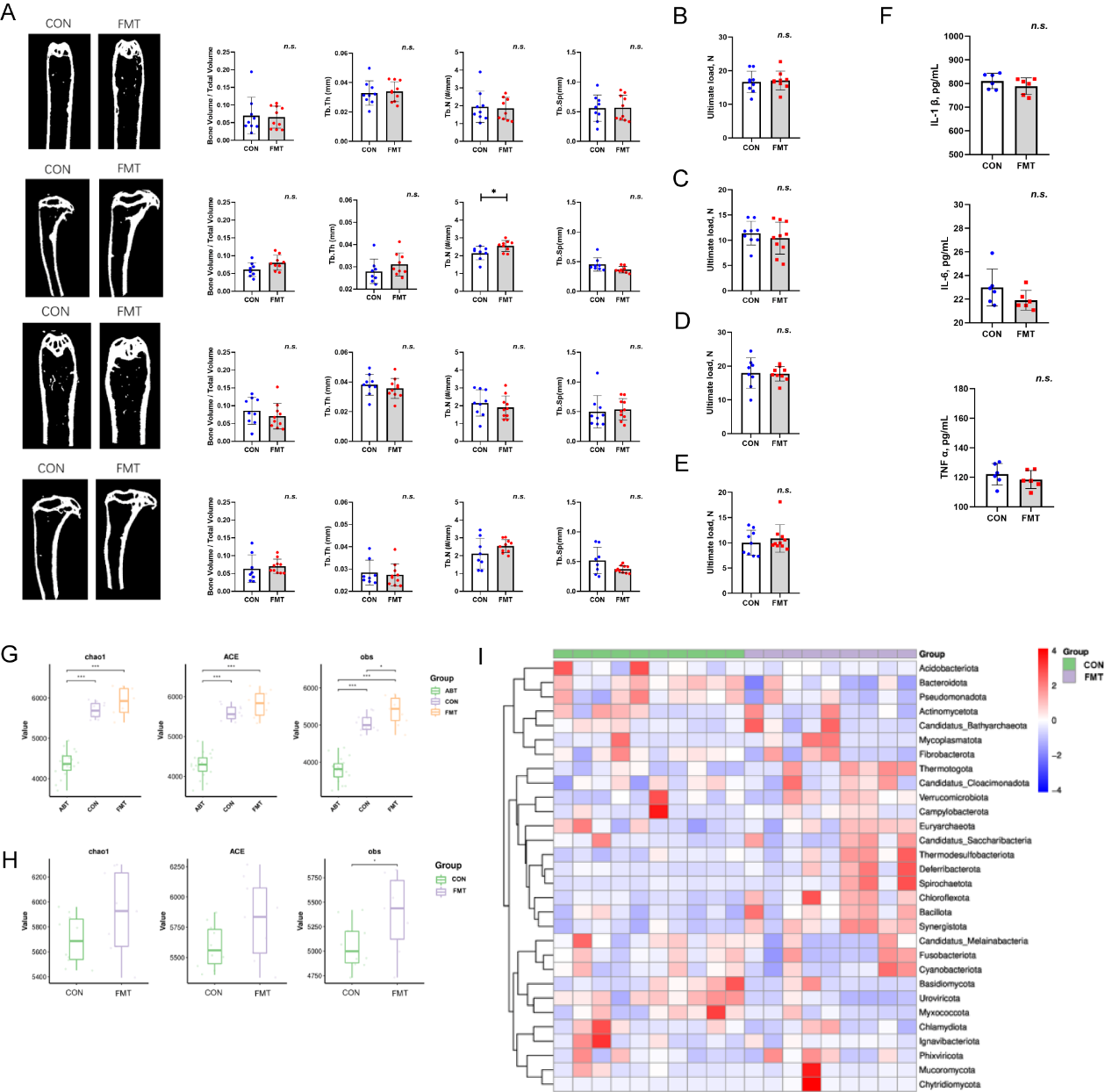


**Figure S4. Bone quality, GM and metabolism of old mice changed after FMT treatment.**

A). Representative µCT images of the three-dimensional trabecular architecture and quantitative analysis of changes in the trabecular microarchitecture of the left femur, left tibia, right femur, right tibia from CON group and FMT group.

B-E). Assessment of ultimate force in left femur (B), left tibia (C), right femur (D), right tibia (E) from CON group and FMT group using three-point bending testing. Error bars represent means ± SD. Values of *P* by two-tailed unpaired-samples *t*-test.

F). ELISA analysis of the serum concentrations of inflammatory markers (IL-6, IL-1 β and TNF-α).

G). Alpha diversity (Chao1, ACE index and Observed otus) of the ABT, CON and FMT groups at the gene level.

H). Alpha diversity (Chao1, ACE index and Observed otus) of the CON and FMT groups at the gene level.

I). Heatmap shown the top 30 species abundance in samples from individuals with CON group mice (green) and FMT group mice (purple).


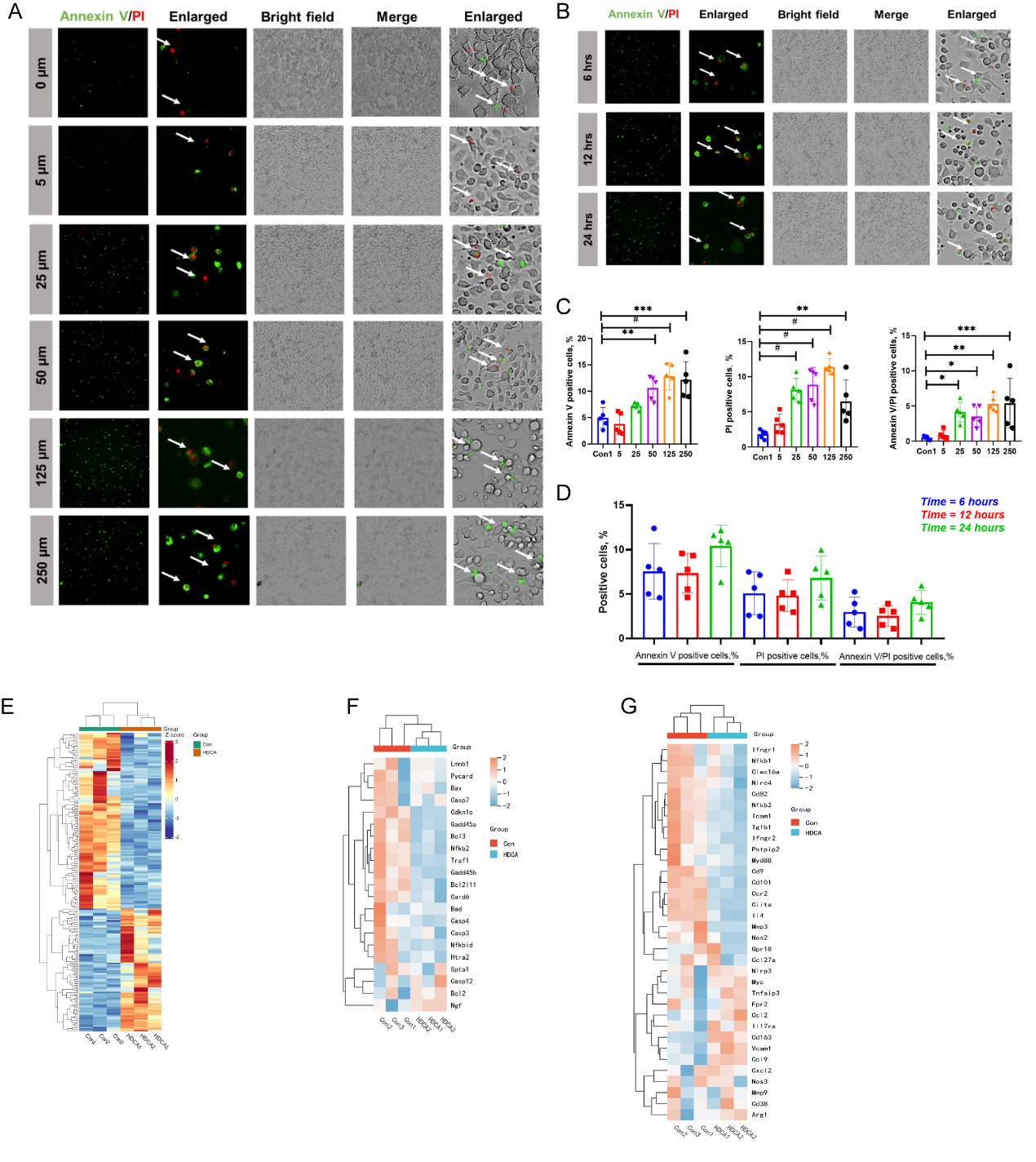


**Figure S5. The effect of HDCA on BMMs.**

A, C). Dual staining with fluorescent Annexin V and PI has been used to discriminate apoptotic and necrotic cell death, in which Annexin V-positive/PI-negative staining is regarded as apoptosis and PI-positive staining as necrosis. Immunofluorescence analysis and the quantification (**C**) of the proportion of Annexin V/PI positive macrophages under different treatment concentrations with 24 hours. *n* = 3 per group

B, D). Immunofluorescence analysis and the quantification (**D**) of the proportion of Annexin V/PI positive macrophages under different treatment times with 50µM HDCA. *n* = 3 per group

E). Heatmap showing difffferentially expressed genes (DEGs) in the HDCA group compared to the control group.

F, G). Heatmap of genes related to apotosis (F) and inflammation (G) in the control and HDCA groups.


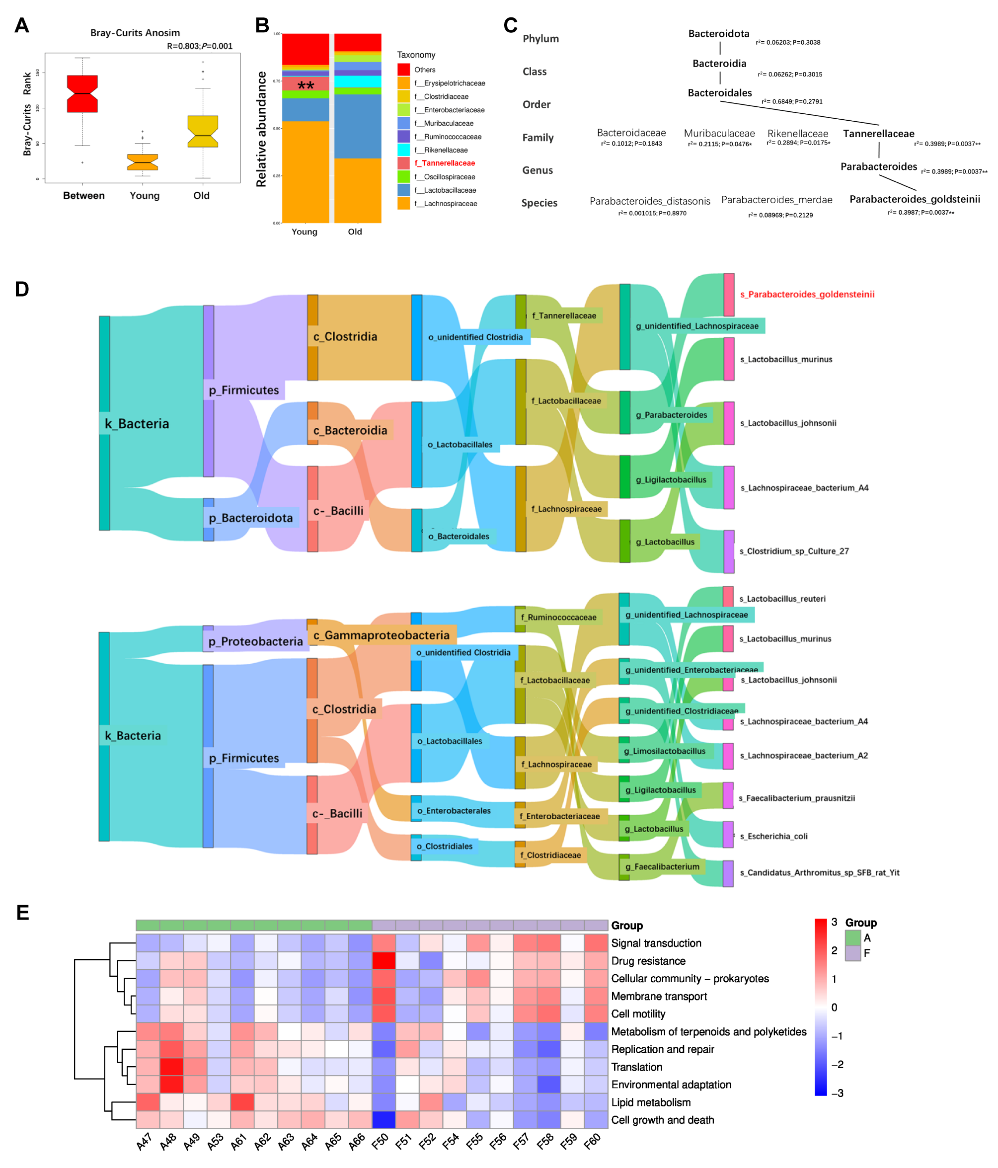


**Figure S6. GM alterations in senile osteoporosis and old mice after FMT.**

A). Bray–Curtis dissimilarity between young and old groups.

B). Composition of GM at the family levels tested by 16S rRNA gene sequencing. n = 9-10 per group. As for family, the proportion <1% occupancy is noted as others. T-test analysis of differences in the relative abundance of species between groups based on OTU showed that *Tannerellaceae* (***P*<0.01) was significant difference between two groups.

C).Pearson’s correlation analyses were used to examine further relationships between the *Bacteroidota* family of bacteria (sub-phylum) populations and BV/TV.

D). A Sankey diagram was drawn to visualize the heterochronous change of GM between the young group and old group subjects. The leftmost side represents the GM in phylum level

and the rightmost side represents GM in species level.

E). Heatmap of Top30 abundance of differential function KEGG pathway at level3.

| **Table S2. Characteristics of included participants.** | | |
| --- | --- | --- |
|  | Young | Old |

| **Lifestyle factors** |  |  |
| --- | --- | --- |
| **Drinking (%)** |  |  |
| Never | 95.00 | 77.50 |
| <0.136 standard cups/day^1,2^ | 3.33 | 15.00 |
| >0.136 standard cups/day | 1.67 | 7.50 |
| **Smoking (%)** |  |  |
| Never | 95.00 | 82.50 |
| Current | 5.00 | 17.50 |
| **Exercise (%)** |  |  |
| <3 times/week^3^ | 13.33 | 20.00 |
| 3-7 times/week | 86.67 | 80.00 |
| >7 times/week | 0.00 | 0.00 |
| **Dietary intake of meat (%)** |  |  |
| <3 times/week | 3.33 | 12.50 |
| 3-7 times/week | 71.67 | 77.50 |
| >7 times/week | 25.00 | 10.00 |
| **Dietary intake of dairy products (%)** |  |  |
| <3 times/week | 28.33 | 47.50 |
| 3-7 times/week | 70.00 | 52.50 |
| >7 times/week | 1.67 | 0.00 |
| **Dietary intake of vegetables (%)** |  |  |
| <3 times/week | 3.33 | 0.00 |
| 3-7 times/week | 83.33 | 72.50 |
| >7 times/week | 13.33 | 27.50 |
| **Dietary intake of eggs (%)** |  |  |
| <3 times/week | 10.00 | 10.00 |
| 3-7 times/week | 76.67 | 85.00 |
| >7 times/week | 13.33 | 5.00 |
| **Self-assessment of dietary structure (%)** |  |  |
| Balanced diet | 33.33 | 40.00 |
| Mainly meat | 33.33 | 15.00 |
| Mainly vegetarian | 25.00 | 32.50 |
| Picky eating | 8.33 | 12.50 |
| **Comorbidity (%)** |  |  |
| Anemia | 0.0 | 0.0 |
| Cancer | 0.0 | 0.0 |
| Chronic obstructive pulmonary disease | 0.0 | 0.0 |
| Diabetes | 0.0 | 12.5 |
| Hyperlipidemia | 0.0 | 25.0 |
| Hypertension | 0.0 | 31.3 |
| Rheumatoid arthritis | 0.0 | 0.0 |
| **Medication (%)** |  |  |
| ACE inhibitors | 0.0 | 2.5 |
| Angiotensin receptor blocker | 0.0 | 2.5 |
| Anticoagulant | 0.0 | 0.0 |
| Antidiabetic | 0.0 | 5.0 |
| Antihypertensive | 0.0 | 12.5 |
| Aspirin | 0.0 | 2.5 |
| Calcium channel blockers | 0.0 | 10.0 |
| Estrogen | 0.0 | 0.0 |
| Glucocorticoids | 0.0 | 0.0 |
| NSAIDs | 1.7 | 12.5 |
| Proton pump inhibitor | 0.0 | 2.5 |
| Statin | 0.0 | 10.0 |
| β-Receptor inhibitor | 0.0 | 5.0 |

| 1. A standard glass is defined as 10 grams of pure alcohol, equivalent to a small glass (100 milliliters) of 13% red wine, a can or bottle (375 milliliters) of 3.5% beer, or a glass (30 milliliters) of 40% spirits. 2. For people aged 15 to 39, the recommended daily alcohol intake is 0.136 standard cups. Exceeding this value may pose health risks. 3. Exercise for 20 minutes or more, including gym workouts, slow walking, running, ball games, yoga, etc. |
| --- |
